# Cable-Plate augmentation improves the therapeutic effect of intramedullary nailing for AO/OTA type A2.3 intertrochanteric fractures with large coronal fragments: a double-center retrospective study

**DOI:** 10.3389/fsurg.2026.1649442

**Published:** 2026-05-15

**Authors:** Guofeng Huang, Zhangxin Chen, Ling Yu, Haimeng Chu, Jingteng Chen, Xiaolong Cai, Taoyi Cai, Weichun Guo, Hui Liu

**Affiliations:** 1 Department of Orthopaedic Surgery, The 909th Hospital, School of Medicine, Xiamen University, Zhangzhou, China; 2 Department of Orthopaedic Surgery, Renmin Hospital of Wuhan University, Wuhan, China; 3 Department of 94895 Health Team, Zhangzhou, China

**Keywords:** A2.3 intertrochanteric fracture, coronal fragments, geriatric trauma, intramedullary nailing, proximal femoral nail antirottion (PFNA)

## Abstract

**Background:**

Intramedullary nailing remains the standard treatment for Association Osteosynthesis/Orthopaedic Trauma Association (AO/OTA) 31-A2.3 intertrochanteric fractures, however, there are still high failure rates when managing those with large displaced coronal fragments. This study presents a modified cable-plate augmentation technique for optimizing coronal fragment stabilization during proximal femoral nail antirotation (PFNA) fixation.

**Methods:**

We conducted a double-center retrospective cohort study of geriatric patients diagnosed with AO/OTA type A2.3 intertrochanteric fractures combined with large displaced coronal fragments, who underwent either standard PFNA fixation or modified cable-plate augmentation combined with PFNA fixation between January 2023 and March 2025. These two cohorts were compared in terms of surgical parameters and complication profiles.

**Results:**

84 geriatric patients with type A2.3 intertrochanteric fractures with displaced large coronal fragments were analyzed. 40 patients received the conventional fixation protocol, and 44 received the augmented fixation protocol. The cohorts demonstrated comparable demographics in terms of age, comorbidity index and bone mineral density. The operative outcomes revealed prolonged surgical duration in the augmented fixation group (86.6 ± 16.9 min vs. 69.7 ± 14.0 min, *p* < 0.01), with higher intraoperative blood loss (134.2 ± 31.6 mL vs. 112.7 ± 43.1 mL, *p* = 0.011). Rehabilitation metrics significantly favored augmented fixation, as indicated by a lower VAS postoperation (3.4 ± 2.1 vs. 4.5 ± 2.3, *p* = 0.026), earlier weight-bearing initiation (2.2 ± 0.8 days vs. 5.1 ± 1.2 days, *p* < 0.01) and accelerated radiographic union (10.5 ± 1.3 weeks vs. 13.2 ± 1.7 weeks,*p* < 0.01). The conventional fixation group presented higher complication rates (20% vs. 4.8%, *p* < 0.05). At the 12-month follow-up, functional recovery was superior in the augmented cohort (HHS: 92.3 ± 12.3 vs. 84.7 ± 13.8, *p* < 0.01) despite equivalent pain scores.

**Conclusion:**

Compared with conventional PFNA fixation, the cable-plate augmentation technique significantly enhances the fixation stability in type A2.3 intertrochanteric fractures with large coronal fragments, resulting in a reduction in complications, acceleration of fracture union, and improvement in functional outcomes.

## Introduction

The global age-standardized incidence rate (ASIR) of geriatric hip fractures rose substantially from 781.56 to 948.81 per 100,000 population between 1990 and 2021. This rise is driven primarily by population aging, with falls constituting the predominant direct cause (1). Proximal femoral nail antirotation (PFNA) has emerged as the predominant surgical intervention for geriatric intertrochanteric fractures, demonstrating favorable clinical outcomes through closed reduction techniques (2). However, reported failure rates of 31% persist, which is predominantly attributable to fracture displacement following mobilization (3). The sliding compression mechanism of PFNA paradoxically increases instability risks in fractures with compromised lateral wall integrity or posteromedial comminution, where excessive fragment mobility elevates the risks of implant cut-out and secondary displacement (4), particularly when early weight-bearing is pursued, which is the key therapeutic goal in elderly patients (5).

AO/OTA (Association for the Osteosynthesis/ Orthopaedic Trauma Association) A2.3 intertrochanteric fractures represent a distinct clinical entity frequently misclassified as simple fractures owing to subtle coronal plane involvement (6). These fractures typically present with displaced coronal fragments (Cho JW classification type 3) (6), characterized by bone segments extending from the greater trochanter apex to the lesser trochanter base (7). This morphology dually disrupts posteromedial cortical support and partial lateral wall stability (8), creating a biomechanical environment predisposed to nonunion, screw migration, and varus collapse with conventional PFNA fixation (9). The unaddressed displacement of lesser trochanter fragments bearing iliopsoas attachments further compromises hip flexion mechanics and promotes progressive coxa vara (10).

Current adjunctive strategies for coronal fragment stabilization, including supplemental plate and cerclage wiring, either increase surgical trauma or achieve suboptimal fixation strength, demonstrating limited efficacy (4, 11). This study introduces a minimally invasive cable-plate augmentation technique through an auxiliary incision, designed to optimize coronal fragment fixation while preserving the biological advantages of PFNA. Our protocol aims to reconcile the conflicting demands of anatomical stability and early rehabilitation in this challenging fracture type.

## Methods

### Research protocol and demographic data

**Figure 1:**
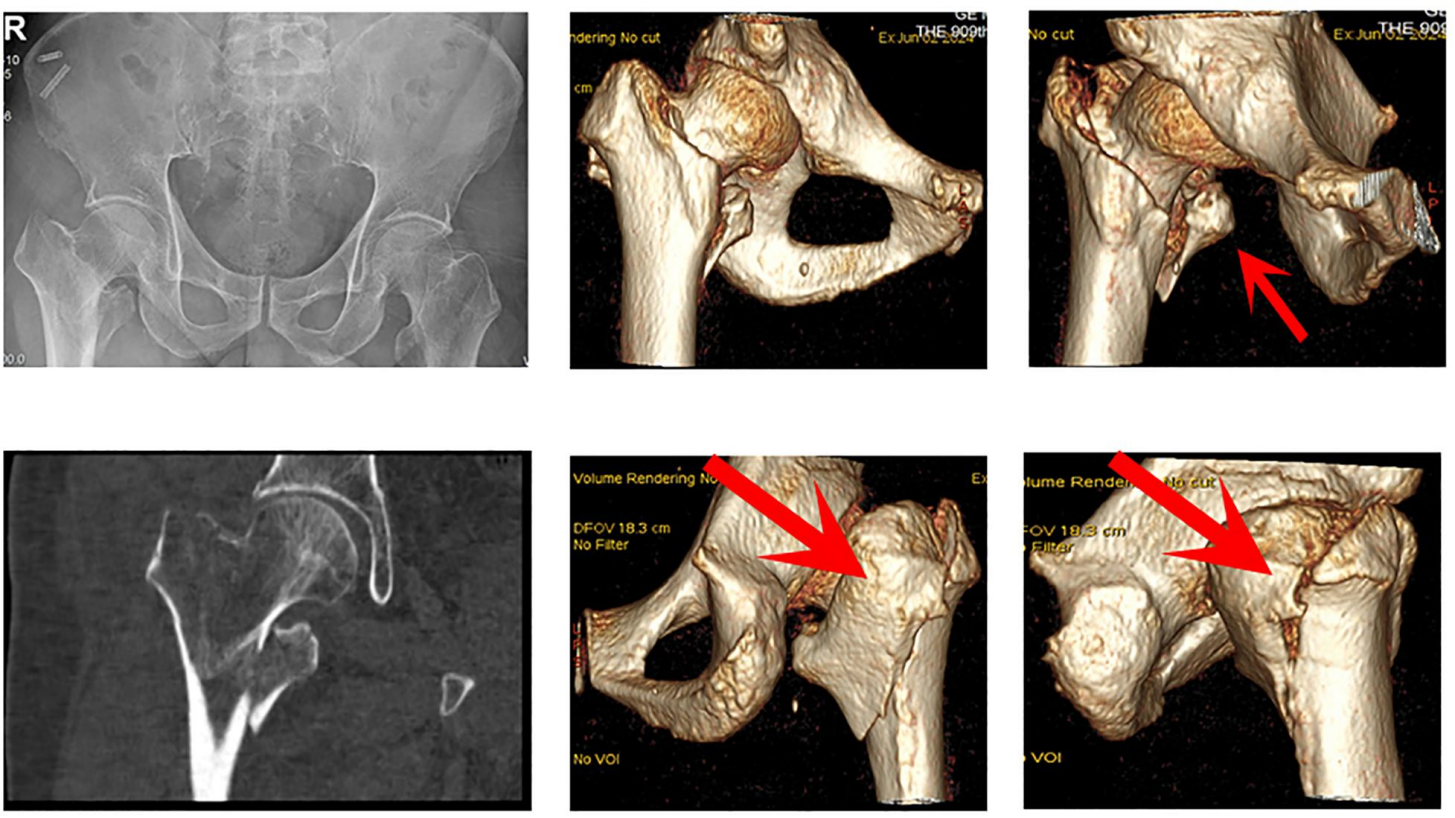
AO/OTA 31-A2.3 intertrochanteric fracture with type 3 large coronal fragments (arrows).

This double-center retrospective study consisted of elderly patients (aged ≧65y) with AO/OTA 31-A2.3 intertrochanteric fractures combined with large displaced coronal fragments (Cho JW classification, Type 3, Figure 1) who underwent surgical treatment at our medical center between January 2023 and March 2025 and completed at least 12 months of follow-up The patients were divided into two groups according to the surgical method: the conventional PFNA group (*n* = 40) and the PFNA combined with cable-plate augmentation group (*n* = 44). The choice of surgical technique was made by the patients themselves after being fully informed of the potential benefits and risks of each option, under the guidance of the attending surgeons. Due to the retrospective nature of this study, no randomization or propensity score matching was conducted. In future work, we plan to conduct a prospective, multicenter study to provide more robust validation of these findings.

### Surgical techniques

**Figure 2:**
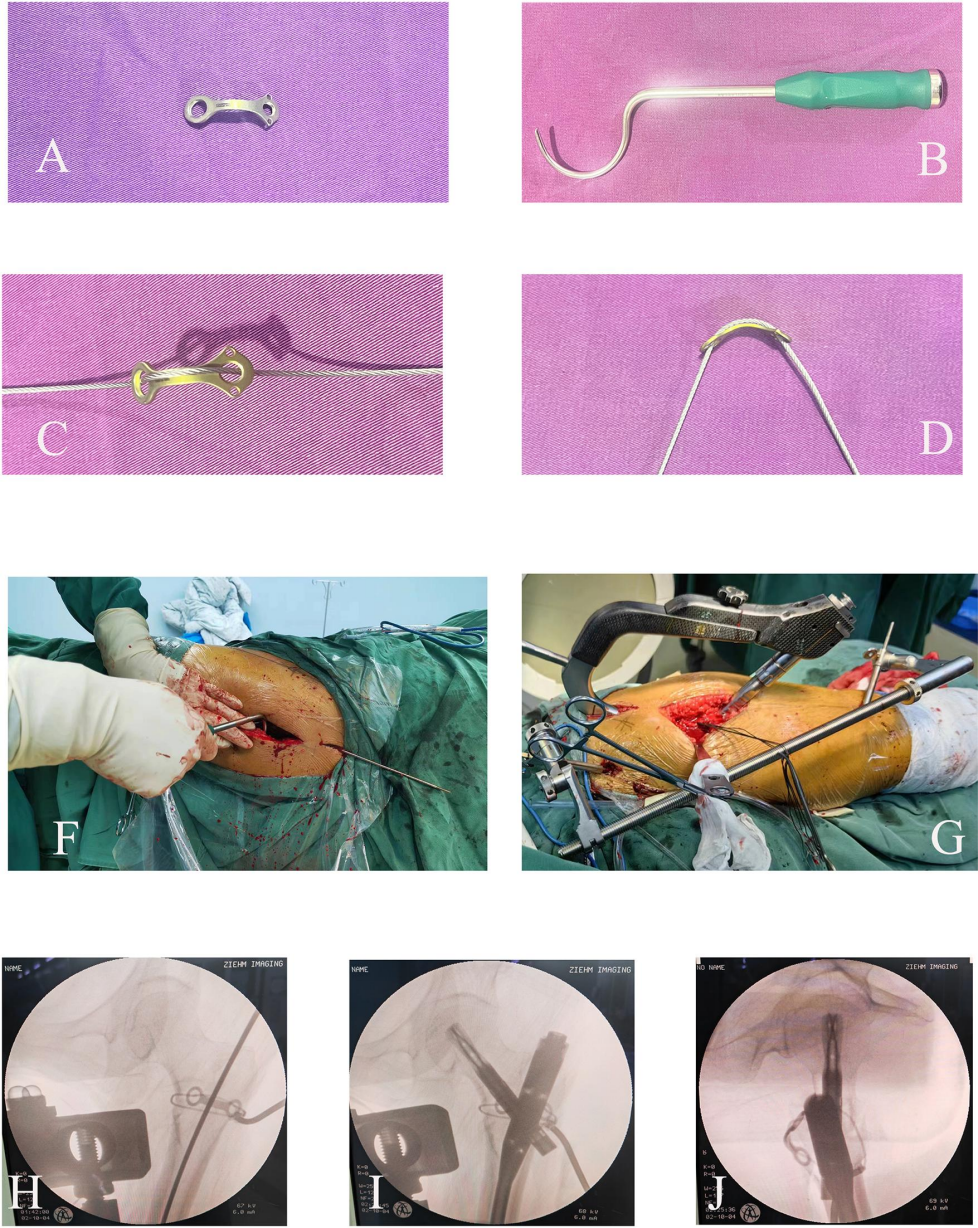
Surgical workflow of the modified cable-plate augmentation technique for PFNA fixation. **(A)** Small precontoured anatomical plate; **(B)** Minimally invasive tools for cable insertion; **(C,D)** Titanium cables combined with an anatomically contoured plate; **(E,F)** Supine positioning on a traction table or in lateral decubitus position with a traction device for reduction, and a minimally invasive approach through the spiral blade entry site incision. **(G)** Anatomical reduction and fixation of large coronal fragments; **(H,I)** Percutaneous PFNA implantation.

The conventional fixation group underwent standard PFNA (DePuy Synthes, USA) implantation via closed reduction either on a traction table or in the lateral decubitus position. For the augmented fixation group, a refined surgical protocol was implemented: After closed reduction with traction, through the incision for the spiral blade (with appropriate enlargement), a hollow wire guide was used to place the titanium cable (Double Medical, China) at the proper position. The cable was passed through the plate (arc-shaped, two-hole compression plate, Double Medical, China) to form an integrated structure. The plate was percutaneously pushed to the posterior side of the coronal bone fragment. A one-way tensioner was used to tighten the titanium cable, and lateral fluoroscopy was performed to observe the reduction degree of the bone fragment. When the reduction was satisfactory, the cable buckle was tightened, and finally a standard PFNA was inserted. (Figure 2) Preoperative 3D-CT reconstruction guided plate customization to match individual fracture morphologies.

### Postoperative management

All patients were routinely given prophylactic antibiotics and anti-osteoporosis treatment following the standard management principles. The postoperative rehabilitation protocol followed a jointly established postoperative rehabilitation procedure. For patients with satisfactory fracture reduction and adequate posteromedial support after surgery, full weight-bearing was permitted immediately after the operation. In contrast, for patients with comminution at the fracture site and insufficient posteromedial support, partial weight-bearing was recommended initially. The weight-bearing plan was further adjusted according to the patient's pain tolerance, as reflected by the VAS score during the first attempt at mobilization.

### Outcome assessment

Operative parameters including surgical duration and intraoperative blood loss were recorded. Postoperative rehabilitation milestones (time to weight-bearing initiation and radiographic union) were monitored through scheduled follow-ups. Complications including surgical site infection, implant failure (screw cut-out or back-out), and nonunion were documented. Functional outcomes were evaluated at 12 months using the Harris hip score. The visual analog scale (VAS) score for pain assessment was recorded on postoperative day 1 and at the 12-month follow-up. Radiographic union was defined as bridging callus formation across three cortical planes on anteroposterior and lateral views.

### Statistical analysis

Continuous variables were assessed for normality using the Shapiro–Wilk test and are presented as means ± standard deviations. Those satisfying normality assumptions were analyzed using independent Student's *t*-tests. Categorical variables were compared using the *χ*^2^-test; however, Fisher's exact test was applied when expected cell frequencies were less than 5. Statistical significance was defined as a two-tailed *p* value < 0.05. All analyses were conducted using SPSS version 26.0, with effect sizes reported as 95% confidence intervals. Because of the retrospective design, no *a priori* sample size calculation was performed. A *post hoc* power analysis was conducted using IBM SPSS Statistics based on the primary outcome, the Harris hip score at 12-month follow-up (92.3 ± 12.3 in the augmented fixation group vs. 84.7 ± 13.8 in the conventional group). Under a two-tailed independent-samples *t*-test (*α* = 0.05, *n*_1_ = 40, *n*_2_ = 44), the analysis demonstrated a large effect size (Cohen's *d* = 1.254) and an achieved power of 1.000, indicating that the current sample size was fully sufficient to detect clinically meaningful between-group differences.

## Results

### Demographics and clinical characteristics

This institutional review board-approved retrospective study analyzed 84 geriatric patients with AO/OTA 31-A2.3 intertrochanteric fractures complicated by type 3 coronal fragments from 90 consecutive cases treated between January 2023 and March 2025. Six patients were excluded because they were lost to follow-up due to changes in their addresses or unreachable phone numbers. The cohort comprised two matched groups: 40 patients who underwent conventional close reduction and PFNA fixation, and 44 who received cable-plate augmentation and PFNA fixation. All patients were alive at the 12-month postoperative follow-up, and the survival rate was 100% in both groups. Demographic analysis revealed comparable baseline characteristics between the two groups, with mean ages of 74.2 ± 8.5 years (range 65–89) in the augmented cohort and 75.9 ± 9.2 years (67–95) in the conventional control control (*p* = 0.384). There were no significant differences in sex distribution, bone mineral density (T-score: −1.87 ± 1.47 vs. −1.94 ± 1.23, *p* = 0.816), or preoperative delay (33.9 ± 8.1 h vs. 36.4 ± 10.0 h, *p* = 0.211). (Table 1).

**Table 1:** Comparison of demographic and clinical characteristics of patients treated with conventional fixation and augmented fixation.

Variable	Conventional fixation group (*n* = 40)	Augmented fixation group (*n* = 44)	*P* value
Demographics			
Sex			0.950
Male	17	19	
Female	23	25	
Age (yr)	75.9 ± 9.2	74.2 ± 8.5	0.384
Clinical characteristics			
Admission-to-surgery time (h)	36.4 ± 10.0	33.9 ± 8.1	0.211
Bone mineral density	−1.94 ± 1.23	−1.87 ± 1.47	0.816
Comorbidities			0.958
1 comorbidity	11	13	
2 comorbidities	7	7	
≥3 comorbidities	4	5	

Fisher's exact test used for sex, comorbidities; *χ*^2^ test used otherwise.

### Outcome assessment

**Figure 3:**
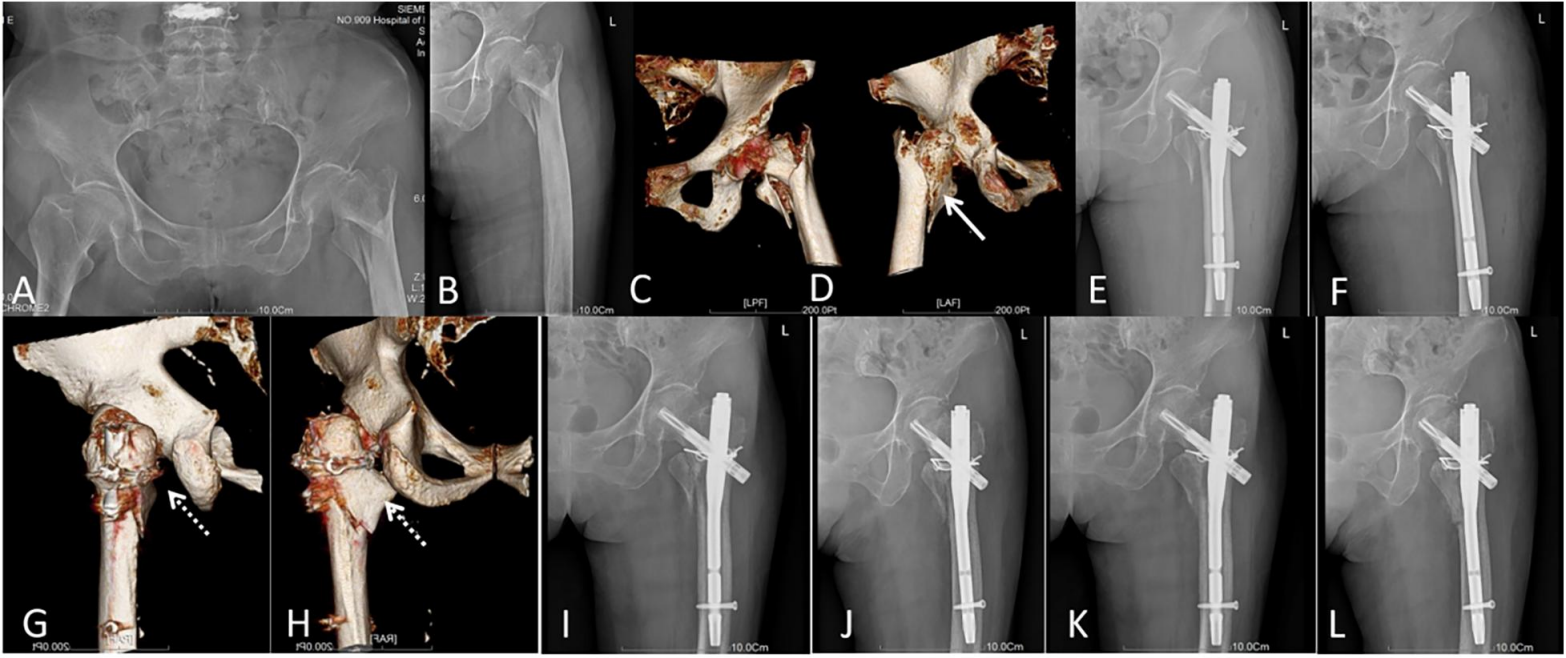
Augmented fixation case: A 73-year-old female sustained a left A2.3 intertrochanteric fracture with type 3 coronal fragments **(A–D)** following a ground-level fall. The patient underwent cable-plate augmentation combined with PFNA fixation **(E–H)**. Weight-bearing ambulation with walker assistance commenced on postoperative day 2. Two-month postoperative radiographs revealed abundant callus formation at the fracture site **(I,J)**, with complete fracture union confirmed at the 12-month follow-up **(K,L)**. The solid arrows delineate the large displaced coronal fragment, whereas the dashed arrows indicate the cable-plate augmentation achieving anatomical reduction and stable fixation.

Operative and postoperative outcomes are summarized in Table 2. The perioperative outcomes demonstrated increased surgical duration for augmented fixation (86.6 ± 16.9 min vs. 69.7 ± 14.0 min, *p* < 0.01) with higher intraoperative blood loss (134.2 ± 31.6 mL vs. 112.7 ± 43.1 mL, *p* = 0.011). The augmented fixation group achieved superior rehabilitation outcomes, including lower postoperative VAS scores (3.4 ± 2.1 vs. 4.5 ± 2.3, *p* = 0.026), earlier postoperative weight-bearing mobilization (2.2 ± 0.8 days vs. 5.1 ± 1.2 days, *p* < 0.01) and accelerated fracture consolidation (10.5 ± 1.3 weeks vs. 13.2 ± 1.7 weeks, *p* < 0.01) (Table 2, Figure 3).

**Table 2:** Comparison of intraoperative and postoperative characteristics of patients treated with conventional fixation and augmented fixation.

	Conventional fixation group (*n* = 40)	Augmented fixation group (*n* = 44)	*P* value
Operative data			
Operative duration (min)	69.7 ± 14.0	86.6 ± 16.9	<0.01
Blood loss (mL)	112.7 ± 43.1	134.2 ± 31.6	0.011
Postoperative outcomes			
VAS (Day1 postoperation)	4.5 ± 2.3	3.4 ± 2.1	0.026
Time to weight-bearing (d)	5.1 ± 1.2	2.2 ± 0.8	<0.01
Fracture healing time (wk)	13.2 ± 1.7	10.5 ± 1.3	<0.01
Harris hip score (1 year follow-up)	84.7 ± 13.8	92.3 ± 12.3	<0.01
VAS (1 year follow-up)	1.6 ± 0.8	1.5 ± 0.7	0.562
Complications			0.042
Overall	8	2	
Nail migration	3	1	
Screw cut-out	2	0	
Nonunion	3	1	

Fisher's exact test used for Complications; *χ*^2^-test used otherwise.

**Figure 4:**
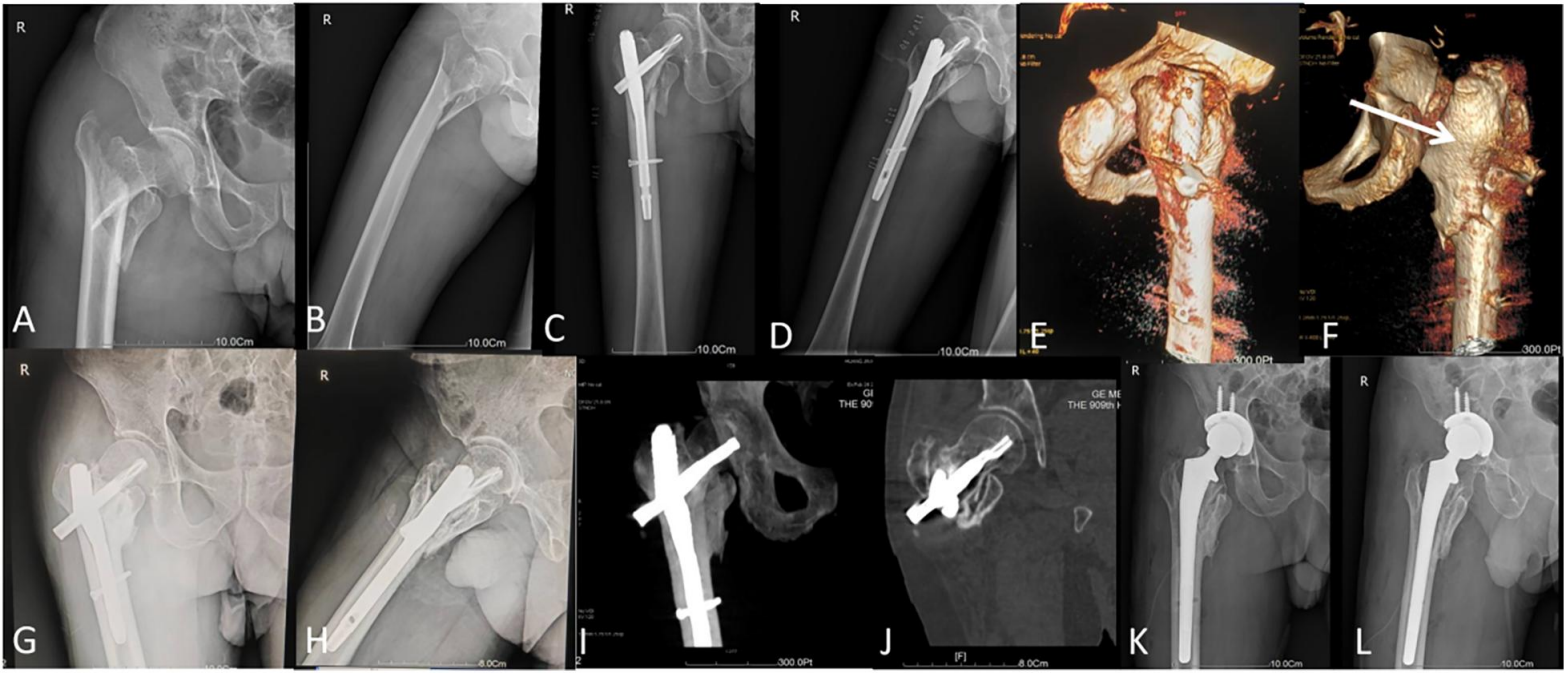
Conventional fixation case: A 68-year-old male presented with a right A2.3 intertrochanteric fracture complicated by type 3 coronal fragments **(A,B)** following low-energy trauma. Conventional closed reduction with PFNA was performed via fracture table traction **(C,D)**. Postoperative 3D-CT reconstruction revealed inadequate coronal fragment stabilization **(E,F)**, and the arrows indicating posteriorly displaced coronal fracture fragments and suboptimal implant entry through the fracture line. At the 12-month follow-up, radiographic evidence revealed nonunion with catastrophic implant fracture **(G–J)**, resulting in revision surgery with conversion to total hip arthroplasty **(K,L)**.

Complication analysis revealed a significant reduction in mechanical failure with augmentation (4.8% vs. 20.0%, *p* < 0.05). Specific complications in the conventional fixation group included 3 cases of screw back-out, 2 cases of cut-out, and 2 cases of nonunion (Figure 4), whereas the augmented group had 1 cases of back-out and 1 cases of nonunion. At the 12-month follow-up, functional recovery significantly favored the augmented cohort (Harris hip score: 92.3 ± 12.3 vs. 84.7 ± 13.8, *p* < 0.01), whereas pain assessment revealed similar outcomes (VAS score: 1.5 ± 0.7 vs. 1.6 ± 0.8, *p* = 0.562) (Table 2).

## Discussion summarized

AO/OTA 31-A2.3 intertrochanteric fractures with large displaced coronal fragments [“banana-shaped fragments” (12, 13)] represent a distinct fracture subtype with unique biomechanical implications (7). The large coronal fragment compromises two critical anatomical regions: first, anterior extension disrupts the lateral femoral wall, which provides natural support for head-neck fragment fixation; second, posterior-inferior extension damages the posteromedial cortex and femoral calcar, which are essential structures for postoperative mechanical load-sharing (14). In elderly patients with AO/OTA 31-A2.3 intertrochanteric fractures, especially those accompanied by large coronal fragments, the biomechanical concept underlying PFNA fixation is that controlled sliding of the helical blade within the nail allows progressive axial compression of the head–neck fragment against the shaft. This dynamic load-sharing mechanism is intended to generate sustained compressive stress at the fracture interface, stimulate callus formation, and preserve the biological environment. However, when a large “banana-shaped” fragment disrupts both the lateral wall and the posteromedial buttress, the same sliding motion is no longer purely axial and controlled, but is redirected into shear and rotational instability. This shift in load transfer predisposes the construct to the failure modes observed in our conventional PFNA cohort.

In patients treated conventionally with closed reduction and PFNA fixation, two predominant failure mechanisms are observed. First, an entry point traversing the fracture line combined with compromised posteromedial support induces sagittal plane instability. This manifests as the “windshield-wiper effect,” leading to subsequent displacement or nonunion (15). Second, unattached coronal fragments—particularly those involving the gluteus medius insertion [posterior greater trochanter (16)], quadratus femoris attachment (intertrochanteric crest), and iliopsoas insertion (lesser trochanter)—may cause pain during hip motion and functional impairment. Collectively, these factors heighten the risk of early weight-bearing failure. This is evidenced by a 20% implant failure rate in our conventional PFNA cohort, a finding consistent with prior literature.

Recent advancements in proximal augmentation techniques aim to address the risk of intramedullary nail failure in A2.3 fractures involving coronal fragments (17). Current strategies include static interlocking nails (e.g., InterTAN) (18), supplemental posterior buttress plates (19), greater trochanter-specific plates (13), and cerclage cabling (20, 21). While InterTAN permits closed implantation, large-scale studies indicate that for unstable patterns, it can still present implant-related failure rates ranging from 4% to 8%, which contributes to higher reoperation rates compared to standard PFNA in some cohorts (22, 23). Similarly, percutaneous cerclage wiring, despite its minimally invasive nature, is associated with a complication risk of cut-through or loss of reduction in 5% to 10% of cases, particularly in osteoporotic bone, as it often fails to maintain sustained compression due to localized pressure concentrations (24, 25). To enhance reduction of large coronal fragments while minimizing these failure risks, we developed our cable-plate augmentation technique. Compared with the existing strategies described above, the cable-plate–augmented PFNA construct used in the present study provides fragment-specific posterior buttressing through a low-profile plate–cable loop placed directly behind the coronal fragment. This configuration restores medial and posteromedial load-bearing pathways without the extensive soft-tissue dissection and periosteal stripping required for open buttress plating, and it avoids the line-contact, high-pressure characteristics of simple cerclage loops, which can cut through osteoporotic bone and lose reduction. By mechanically integrating the coronal fragment into the load-sharing construct rather than relying solely on the implant–bone interface, the technique achieves both mechanical specificity and biological friendliness, which is consistent with the improved stability, earlier weight-bearing, faster fracture union, and superior functional outcomes observed in the augmented group.

A critical consideration from our results is the statistically significant increase in operative time for the cable-plate group (86.6 vs. 69.7 min, *p* < 0.01). It is well-established that prolonged surgical duration can be a risk factor for complications such as surgical site infections (26). However, our comparative analysis did not find an increased infection rate; in fact, the overall complication rate was lower in the augmented group. This suggests that the clinical advantages conferred by the enhanced stability—namely, earlier initiation of weight-bearing, accelerated fracture union, and superior 1-year Harris Hip Scores—effectively offset the potential risks of a modestly extended surgical time. The additional time is directly attributable to the precise placement of the plate and cable, a step that provides the robust fixation necessary to prevent the very mechanical failures that plague traditional methods in this specific, challenging fracture pattern.

This technique confers several clinical advantages. First, it restores posteromedial biomechanical integrity: anatomical reconstruction of the tension-side cortex and calcar region through cable-plate fixation redistributes mechanical loads across both implant and osseous structures, mitigating stress concentration at the nail-bone interface. Second, enhanced proximal femur stability enables accelerated postoperative weight-bearing, facilitating earlier functional recovery. Third, the augmentation system demonstrated significantly lower mechanical failure rates than conventional PFNA, particularly in preventing screw cut-out and back-out complications; this corroborates biomechanical advantages reported in lateral wall reinforcement studies. From a technical perspective, the augmentation procedure is built upon the standard PFNA workflow and therefore imposes a relatively low learning burden on surgeons. Through a modest extension of the helical-blade incision, a pre-contoured mini-arc plate and titanium cable can be advanced percutaneously behind the coronal fragment and tensioned under fluoroscopic guidance. Apart from mastering cable routing and tension adjustment, all other steps are identical to conventional PFNA. With accumulating experience, the additional operative time associated with the augmentation step decreased from approximately 20 min in the initial cases to around 15 min, with only minimal extra blood loss and no observed increase in infection or neurovascular injury. These features suggest that the technique has a short learning curve and is highly reproducible in trauma teams already familiar with PFNA.

There are two inherent limitations of this study. First, this was a retrospective analysis, and the treatment allocation was not randomized, which may be associated with selection bias. Patients with better physical condition and higher treatment compliance might be more likely to choose the modified fixation technique. Nevertheless, the baseline characteristics between these two groups were well matched, suggesting that the observed between-group differences were unlikely to be attributable to measured baseline factors. Second, the sample of enrolled patients was small. Further studies with large research samples and prospective designs with clinical follow-up data would be beneficial for demonstrating more reliable conclusions.

## Conclusion

Compared with conventional PFNA fixation, the cable-plate augmentation technique significantly enhances the fixation stability in geriatric type A2.3 intertrochanteric fractures with large coronal fragments,reducing postoperative complications, accelerating fracture union, and improving functional outcomes.

## Data Availability

The original contributions presented in the study are included in the article/Supplementary Material, further inquiries can be directed to the corresponding author/s.
