# Correction: Cable-Plate augmentation improves the therapeutic effect of intramedullary nailing for AO/OTA type A2.3 intertrochanteric fractures with large coronal fragments: a double-center retrospective study

**DOI:** 10.3389/fsurg.2026.1882493

**Published:** 2026-06-12

**Authors:** Guofeng Huang, Zhangxin Chen, Ling Yu, Haimeng Chu, Jingteng Chen, Xiaolong Cai, Taoyi Cai, Weichun Guo, Hui Liu

**Affiliations:** 1 Department of Orthopaedic Surgery, The 909th Hospital, School of Medicine, Xiamen University, Zhangzhou, China; 2 Department of Orthopaedic Surgery, Renmin Hospital of Wuhan University, Wuhan, China; 3 Department of 94895 Health Team, Zhangzhou, China

**Keywords:** A2.3 intertrochanteric fracture, coronal fragments, geriatric trauma, intramedullary nailing, proximal femoral nail antirottion (PFNA)

There was a mistake in Table 1 as published. The *P* value for Sex was displayed as 1.000 and the *P* value for Comorbidities was displayed as 1.000. The correct *P* value for Sex is 0.950, and the correct *P* value for Comorbidities is 0.958. The corrected Table 1 appears below.

**Table 1:** Comparison of demographic and clinical characteristics of patients treated with conventional fixation and augmented fixation.

Variable	Conventional fixation group (*n* = 40)	Augmented fixation group (*n* = 44)	*P* value
Demographics			
Sex			0.950
Male	17	19	
Female	23	25	
Age (yr)	75.9 ± 9.2	74.2 ± 8.5	0.384
Clinical characteristics			
Admission-to-surgery time (h)	36.4 ± 10.0	33.9 ± 8.1	0.211
Bone mineral density	−1.94 ± 1.23	−1.87 ± 1.47	0.816
Comorbidities			0.958
1 comorbidity	11	13	
2 comorbidities	7	7	
≥3 comorbidities	4	5	

Fisher's exact test used for sex, comorbidities; *χ*^2^ test used otherwise.

The original version of this article has been updated.

